# Inconsistencies in domestic land use change study

**DOI:** 10.1073/pnas.2213961119

**Published:** 2022-12-13

**Authors:** Tania M. Alarcon Falconi, Fatemeh Kazemiparkouhi, Brittany Schwartz, David L. MacIntosh

**Affiliations:** ^a^Environmental Health & Engineering, Inc., Newton, MA 02459; ^b^Tufts University, Boston, MA 02111; ^c^Harvard T.H. Chan School of Public Health, Boston, MA 02115

Lark et al. (1) (hereafter “Lark 2022”) present a corn ethanol domestic land use change (dLUC) study that conflicts with the best available science and, we believe, is based on a flawed corn price analysis.

## Conflicting Characterization of Corn Price and Ethanol Demand.

**Fig. 1.**
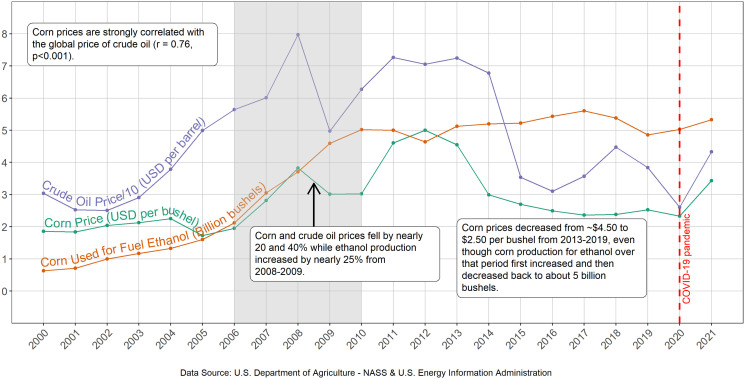
Annual corn and crude oil prices (adjusted with the Consumer Price Index), and corn used for ethanol from 2000 to 2021.

Lark 2022 reports that demand for field corn associated with the 2010 Renewable Fuel Standard (RFS2) caused a persistent 31% corn price increase, representing a 5.6% corn price increase for every billion-gallon increase in ethanol demand. (1) The 2006–2010 period used in Lark 2022 to determine the relationship of corn price with ethanol demand is the only 5-y period since 2006 when the two parameters rose simultaneously (Fig. 1, shaded panel). In contrast, the price of corn and the amount of corn consumed for ethanol production are not correlated from the inception of RFS2 through the present (r = 0.13, *P* = 0.64, 2006–2021). Moreover, the price of corn in recent years prior to market disruptions caused by conflict in Ukraine was approximately the same as during the years that preceded the period of the Lark 2022 analysis. The Lark 2022 analysis appears to have excluded the 10 y of data (2011–2020) that do not support their conclusion. We also note that the price of corn is strongly correlated with crude oil price (r = 0.86 between 1991 and 2016), (2) as shown in the figure from 2000 to 2021, a factor that did not appear to be included in the Lark 2022 analysis.

## Deviation from Accepted dLUC Values.

**Fig. 2.**
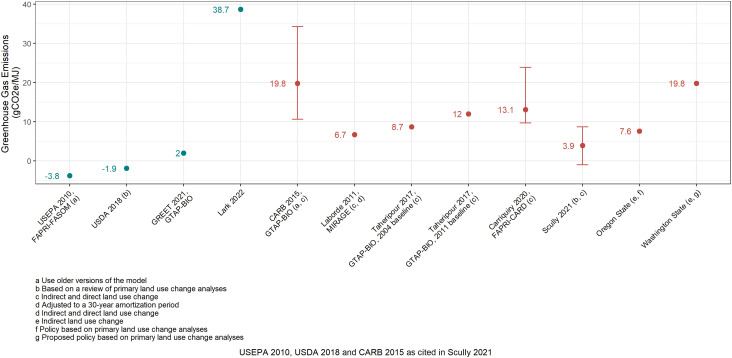
Lark 2022 dLUC result deviates substantially from relevant recent studies of dLUC (teal) and total LUC (red).

Lark 2022 concludes that RFS2 resulted in dLUC impacts of at least 38.7 g of carbon dioxide equivalent emissions per megajoule of ethanol (gCO2e/MJ) with additional unquantified impacts from international land use change (iLUC). The Lark 2022 findings are based in part upon corn prices projected for a counterfactual analysis where RFS2 was not proposed or promulgated. Notably, the projected annual corn prices presented in Lark 2022 are below the cost of corn production published by the U.S. Department of Agriculture in 8 of the 11 y (2006–2016), (3) which was not discussed in the paper. Moreover, Lark 2022 indicates the counterfactual scenario analysis is based on only three crops – corn, soybean, and wheat. In contrast, generally accepted and relied upon models of land cover change, such as GTAP-BIO, FAPRI-CARD, and MIRAGE, simulate economy-wide and global market dynamics to determine land cover responses due to biofuel production. While variation exists among results from those models, which were independently developed in the U.S. and Europe, the latest dLUC estimates are typically within twofold of each other and lower than the Lark 2022 estimate (Fig. 2). (4–10) Study limitations, such as a flawed corn price analysis, reliance on an unrealistic counterfactual scenario, and a narrow focus on three crops may be driving the high dLUC estimate reported in Lark 2022.
